# Donanemab treatment effect by baseline tau burden and disease severity: Observations from the TRAILBLAZER‐ALZ 2 trial

**DOI:** 10.1002/alz.71577

**Published:** 2026-06-11

**Authors:** Lars L. Raket, Ming Lu, Cynthia D. Evans, Jennifer A. Zimmer, JonDavid Sparks, Emily C. Collins, Sergey Shcherbinin, Hong Wang, Emel Serap Monkul Nery, Stéphane Epelbaum, Grazia Dell'Agnello, Dawn A. Brooks, John R. Sims, Mark A. Mintun

**Affiliations:** ^1^ Eli Lilly and Company Indianapolis Indiana USA

**Keywords:** Alzheimer's disease, amyloid‐targeting therapy, disease progression modeling, neurodegeneration, P‐tau217, tau PET

## Abstract

**INTRODUCTION:**

Clinical trials indicate that disease‐modifying therapies can slow clinical decline in Alzheimer's disease (AD), with earlier initiation associated with greater slowing.

**METHODS:**

In the TRAILBLAZER‐ALZ 2 trial, the treatment effect of donanemab on the Clinical Dementia Rating Scale Sum of Boxes (CDR‐SB) score was assessed across disease stages defined by baseline tau PET, plasma P‐tau217 levels, or predicted disease progression.

**RESULTS:**

Donanemab‐mediated slowing of disease progression occurred across baseline tau PET and plasma P‐tau217 levels. Participants with lower baseline tau PET and P‐tau217 showed greater slowing with donanemab versus placebo. Modeling CDR‐SB scores indicated that earlier treatment (at the 25^th^ percentile of baseline Predicted disease progression) delayed disease progression by 60% over 76 weeks, compared to 33% and 17% at the 50^th^ and 75^th^ percentiles.

**DISCUSSION:**

Donanemab benefited participants with early symptomatic AD across clinical and pathological severities, with the greatest slowing in those treated earlier.

ClinicalTrials.gov Identifier: NCT04437511

## Highlights

- Donanemab slowed clinical decline across baseline tau positron emission tomography (PET) and P‐tau217 levels
- Lower baseline tau PET and plasma P‐tau217 was associated with greater slowing
- Earlier treatment delayed disease progression by 60% during the study

## 1 BACKGROUND

Revised criteria for the diagnosis and staging of Alzheimer's disease (AD) emphasize the use of biomarkers for accuracy^1^. Core biomarkers like phosphorylated (P)‐tau217 and tau positron emission tomography (PET) detect early pathological changes and can be used to track disease progression.^1^ Intervention earlier in the clinical and/or neuropathological process is associated with better outcomes.^2^ The TRAILBLAZER‐ALZ (NCT03367403)^3^ phase 2 and TRAILBLAZER‐ALZ 2 (NCT04437511)^2^ phase 3 trials demonstrated that donanemab treatment slows disease progression even more effectively in participants with less advanced disease.

We present post‐hoc analyses exploring the effect of donanemab treatment on slowing clinical decline in TRAILBLAZER‐ALZ 2 participants according to baseline tau PET or plasma P‐tau217 levels and the participant's predicted disease progression derived based on a previously published disease progression modeling framework.^4^

## 2 METHODS

The methods employed in TRAILBLAZER‐ALZ 2 and the baseline characteristics of the trial population have been previously published.^2^ Briefly, TRAILBLAZER‐ALZ 2 was a global, phase 3 clinical trial that enrolled 1736 participants with early symptomatic AD (mild cognitive impairment or mild dementia). The presence of both amyloid and tau pathology was confirmed by PET imaging. Participants were randomized 1:1 to receive donanemab (n  =  860) or placebo (n  =  876) intravenously at 4‐week intervals for 72 weeks. Those in the donanemab group were switched to placebo in a blinded manner if meeting dose completion criteria (amyloid clearance as assessed by PET). Global cortical tau PET levels were measured using flortaucipir with previously published^5^ AD signature–weighted neocortical standardized uptake value ratios (SUVr). Exploratory tau outcomes included plasma P‐tau217 (C_2_N Diagnostics) measurements.

Efficacy was assessed by change from baseline (CFB) in the Clinical Dementia Rating Scale Sum of Boxes (CDR‐SB) score.^6^ The effect of baseline tau PET levels and P‐tau217 levels on donanemab‐mediated slowing of disease progression was measured by the relative reduction in clinical decline in the donanemab group compared to placebo. The interactive effect of baseline tau PET levels on CFB in CDR‐SB was analyzed using the prespecified mixed model for repeated measures.^2^ This included a baseline tau PET SUVr‐by‐visit‐by‐treatment adjustment term added to the prespecified adjustment factors.^2^ For baseline P‐tau217 levels, which had some extreme values, the interaction was assessed by splitting P‐tau217 levels into terciles and including a tercile group‐by‐basis expansion term‐by‐treatment interaction in the natural cubic spline model with two degrees of freedom, along with the prespecified adjustment factors.^2^

The trial was conducted in accordance with the Declaration of Helsinki, the International Conference on Harmonization Good Clinical Practice Guidelines, and local regulatory requirements. An independent ethics committee/institutional review board at each site approved the study. Participants provided written consent, and an independent data and safety monitoring board provided trial oversight.

To explore the relationship between disease stage and slowing of disease progression, a latent‐time mixed‐effects model^4^ was fitted on longitudinal trajectories of CDR‐SB scores. The latent‐time disease progression model assumes that observed participant‐level CDR‐SB trajectories in the placebo group are short‐term (up to 76 weeks) observations of a common long‐term natural history trajectory across the population. The model estimates the natural history trajectory using placebo group data while simultaneously determining where each participant is positioned along this trajectory at study entry—this position on the natural history trajectory is defined as the participant's predicted disease progression. The model thus accounts for the heterogeneity in baseline disease stage by recognizing that participants enter the trial at different points in their disease course.

The donanemab treatment effect was modeled as a proportional slowing of progression (time scaling factor) following donanemab exposure, meaning that donanemab‐treated participants started on the common trajectory, but branched off due to the treatment‐induced slowing of disease progression. Critically, this treatment effect was allowed to vary as a function of baseline disease stage, quantifying how efficacy depends on when treatment is initiated along the disease continuum. By incorporating disease stage‐dependent treatment effects, the model provides a framework for projecting treatment effects beyond the 76‐week trial period that captures the evolving relationship between disease stage and treatment response over time.

**RESEARCH IN CONTEXT**

1. **Systematic review**: The authors reviewed the literature using PubMed. Clinical trials of disease‐modifying therapies have suggested that slowing of clinical decline is possible, and that earlier intervention may result in greater benefit.
2. **Interpretation**: Donanemab substantially slowed clinical decline compared to placebo across baseline tau positron emission tomography (PET) and P‐tau217 levels, with greater slowing observed in those with lower baseline levels. At the earliest clinical and pathological stage, starting donanemab treatment was associated with a 60% delay in clinical decline during the study and projected delays to the onset of severe dementia.
3. **Future directions**: Further studies are needed to expand on these findings; however, this research suggests that early intervention with disease‐modifying therapies should be prioritized.

Different extrapolation scenarios are possible based on the estimated model parameters; here we present a diminishing slowing scenario, where treatment effects gradually attenuate over time. Specifically, after each 76‐week interval, the model recalculates the magnitude of slowing based on the participant's current position on the disease progression timeline, applying the treatment effect associated with initiating treatment at that disease stage. More details on modeling methodology are available in the eMethods in Supplement 1.

Consistent with the predefined analyses of the primary and secondary endpoints in TRAILBLAZER‐ALZ 2, all models addressed attrition using a hypothetical strategy, which allows us to include all available data without imputing missing values. This approach assumes that data are missing at random, meaning that the likelihood of dropout is unrelated to unobserved outcomes after accounting for observed data. Under this assumption, the model estimates what treatment effects would look like if all participants had remained in the study, with treatment adherence similar to the participants who did not drop out.

## 3 RESULTS

A total of 1736 participants with early symptomatic AD (mild cognitive impairment or mild dementia) were randomized 1:1 to donanemab (n  =  860) or placebo (n  =  876) as previously described.^2^ A total of 794 participants in the donanemab group and 838 participants in the placebo group had a valid CDR‐SB assessment at baseline and one or more post‐baseline (Supplement 2, eFigure 1). Baseline demographics and clinical characteristics split by plasma P‐tau217 terciles are shown in Table 1 and by tau PET categories in Supplement 3, eTables 1 and 2.

**TABLE 1:** Baseline demographics and clinical characteristics

Parameter	Tercile 1 (*N* = 545)	Tercile 2 (*N* = 546)	Tercile 3 (*N* = 545)	Total (*N* = 1636)
Age, mean [SD], y	72.73 [5.96]	73.45 [6.15]	72.72 [6.49]	72.97 [6.21]
Age category, No. (%)				
< 65	50 (9.2)	52 (9.5)	68 (12.5)	170 (10.4)
65‐74	279 (51.2)	237 (43.4)	252 (46.2)	768 (46.9)
≥75	216 (39.6)	257 (47.1)	225 (41.3)	698 (42.7)
Sex, No. (%)				
Female	284 (52.1)	302 (55.3)	349 (64.0)	935 (57.2)
Male	261 (47.9)	244 (44.7)	196 (36.0)	701 (42.8)
Race, No. (%)				
American Indian or Alaska Native	1 (0.2)	0	1 (0.2)	2 (0.1)
Asian	17 (3.1)	45 (8.3)	41 (7.5)	103 (6.3)
Black or African American	15 (2.8)	15 (2.8)	5 (0.9)	35 (2.1)
White	512 (93.9)	484 (88.8)	498 (91.4)	1494 (91.4)
Multiple	0	1 (0.2)	0	1 (0.1)
Missing	0	1	0	1
Ethnicity, No. (%)^a^				
Hispanic or Latino	32 (7.8)	15 (3.9)	23 (6.0)	70 (5.9)
Not Hispanic or Latino	380 (92.2)	370 (96.1)	358 (94.0)	1108 (94.1)
Missing	1	2	0	3
Time since symptom onset, mean [SD], y	3.91 [2.55]	3.78 [2.20]	3.95 [2.38]	3.88 [2.38]
Missing	0	1	0	1
Time since AD diagnosis, mean [SD], y	1.35 [1.87]	1.44 [1.64]	1.55 [1.81]	1.44 [1.78]
Missing	3	5	5	13
*APOE* ε4 status, No. (%)				
Carrier	404 (74.5)	384 (70.3)	358 (65.9)	1146 (70.3)
Non‐carrier	138 (25.5)	162 (29.7)	185 (34.1)	485 (29.7)
Missing	3	0	2	5
Baseline CDR‐SB score, mean [SD]	3.49 [1.93]	3.79 [2.08]	4.42 [2.00]	3.90 [2.04]
Missing	6	6	5	17
Baseline CDR global score, No. (%)				
0	2 (0.4)	2 (0.4)	1 (0.2)	5 (0.3)
0.5	378 (70.1)	351 (65.0)	272 (50.4)	1001 (61.8
1	148 (27.5)	171 (31.7)	249 (46.1)	568 (35.1)
2	11 (2.0)	16 (3.0)	18 (3.3)	45 (2.8)
Missing	6	6	5	17
Baseline MMSE total score^b^, mean [SD]	23.27 [3.57]	22.65 [3.77]	21.19 [3.88]	22.37 [3.84]
Missing	3	3	7	13
Screening tau MUBADA SUVr, mean [SD]	1.21 [0.17]	1.32 [0.22]	1.50 [0.28]	1.34 [0.26]
Missing	31	31	35	97
Screening tau category, No. (%)				
High	78 (14.3)	156 (28.6)	282 (51.9)	516 (31.6)
Intermediate	467 (85.7)	390 (71.4)	261 (48.1)	1118 (68.4)
Missing	0	0	2	2
Screening amyloid centiloid, mean [SD]	98.84 [35.11]	102.26 [33.92]	106.81 [33.59]	102.63 [34.35]

Abbreviations: AD, Alzheimer's disease; APOE, apolipoprotein E; CDR, Clinical Dementia Rating Scale; MMSE, Mini‐Mental State Examination; MUBADA, multi‐block barycentric discriminant analysis; N, number of randomized subjects; SB, Sum of Boxes; SUVr, standardized uptake value ratio.

^a^ Responses only from US/Puerto Rico sites. *n* = 412 tercile 1, *n* = 385 tercile 2, *n* = 381 tercile 3 and *n* = 1178 total.

^b^ Last non‐missing MMSE prior to or on start of study treatment.

In variables with missing data, number of subjects with non‐missing data was used as denominator.

**FIGURE 1 alz71577-fig-0001:**
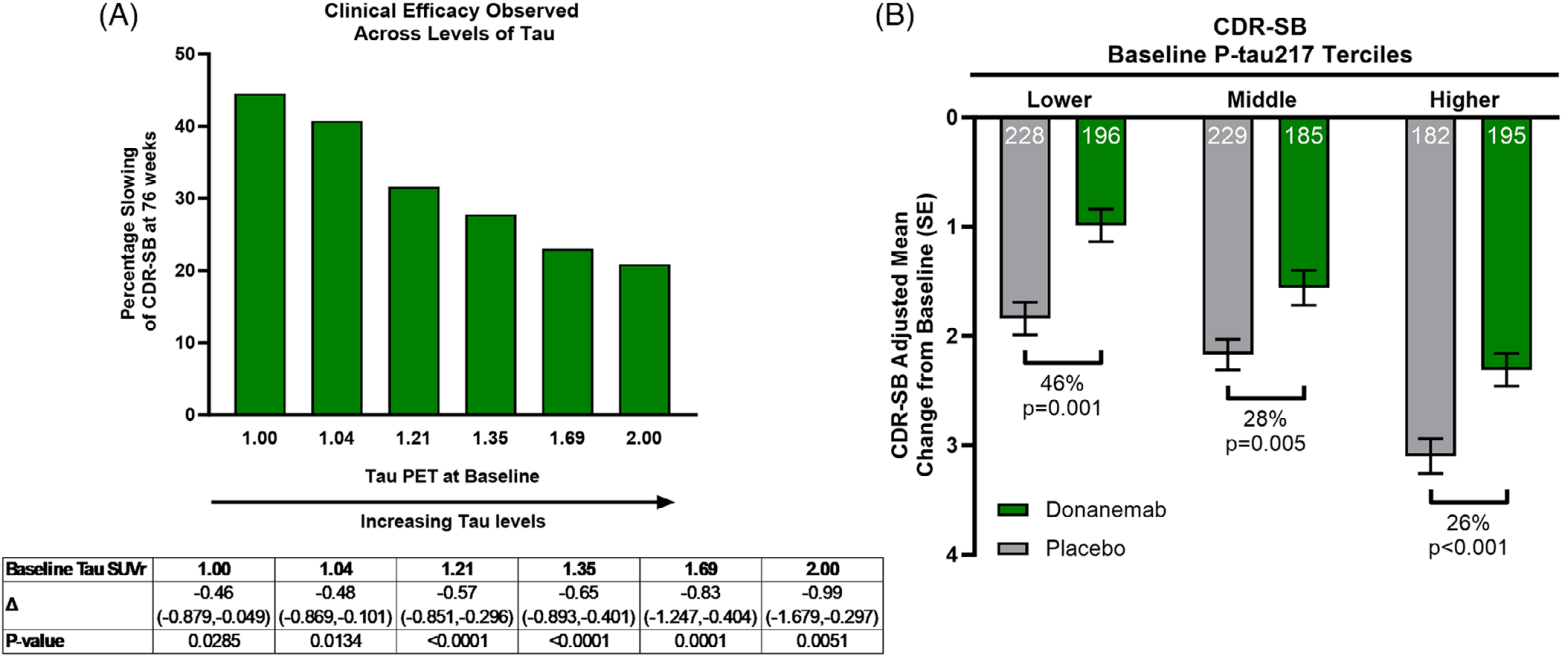
Clinical efficacy estimated across levels of tau: Earlier stage of disease defined by tau biomarkers shows larger donanemab treatment effects compared to placebo. (A) Clinical efficacy estimated across levels of tau. Tau PET categories at baseline: 1.00: anchor point representing no tau; 1.04: historical level of αβ positive, deemed visually tau negative; 1.21: mean in TRAILBLAZER‐ALZ 2 low–medium tau population; 1.35: mean in TRAILBLAZER‐ALZ 2 combined population; 1.69: mean in TRAILBLAZER‐ALZ 2 high tau population; 2.00: anchor point representing very high tau level. The Y‐ axis denotes the percentage slowing of CDR‐SB at 76 weeks. (B) CDR‐SB baseline P‐tau217 terciles. Number of participants are represented within the bars. Adjusted change from baseline, SE, and p‐values are derived using a mixed‐effects model using the natural cubic spline with two degrees of freedom methodology that was prespecified for primary efficacy analyses but including an adjustment term modeling tercile group‐by‐basis expansion term‐by‐treatment interaction. TRAILBLAZER‐ALZ 2 combined population. *p*‐Values are nominal. CDR‐SB, Clinical Dementia Rating Scale Sum of Boxes; SE, standard error.

Compared to placebo, donanemab treatment slowed clinical decline across all baseline tau PET levels, with lower baseline levels associated with a greater percentage slowing of decline in CDR‐SB at 76 weeks compared to placebo (Figure 1A). Correspondingly, higher baseline tau levels were associated with a smaller percentage of slowing.

Figure 1B illustrates the effect of donanemab treatment across baseline P‐tau217 levels, with slowing of clinical decline in those treated with donanemab across all three terciles compared to placebo. Lower baseline P‐tau217 levels were associated with a greater percentage slowing of decline in CDR‐SB at 76 weeks compared to placebo. Donanemab‐treated participants in the lowest tercile (≤33^rd^ percentile) of baseline P‐tau217 levels exhibited 46% slowing of disease progression compared to the placebo group. Donanemab‐treated participants in the middle tercile (33^rd^–66^th^ percentile) had 28% slowing, and in the higher tercile (> 66^th^ percentile), donanemab‐treated participants showed 26% slowing compared to placebo.

**FIGURE 2 alz71577-fig-0002:**
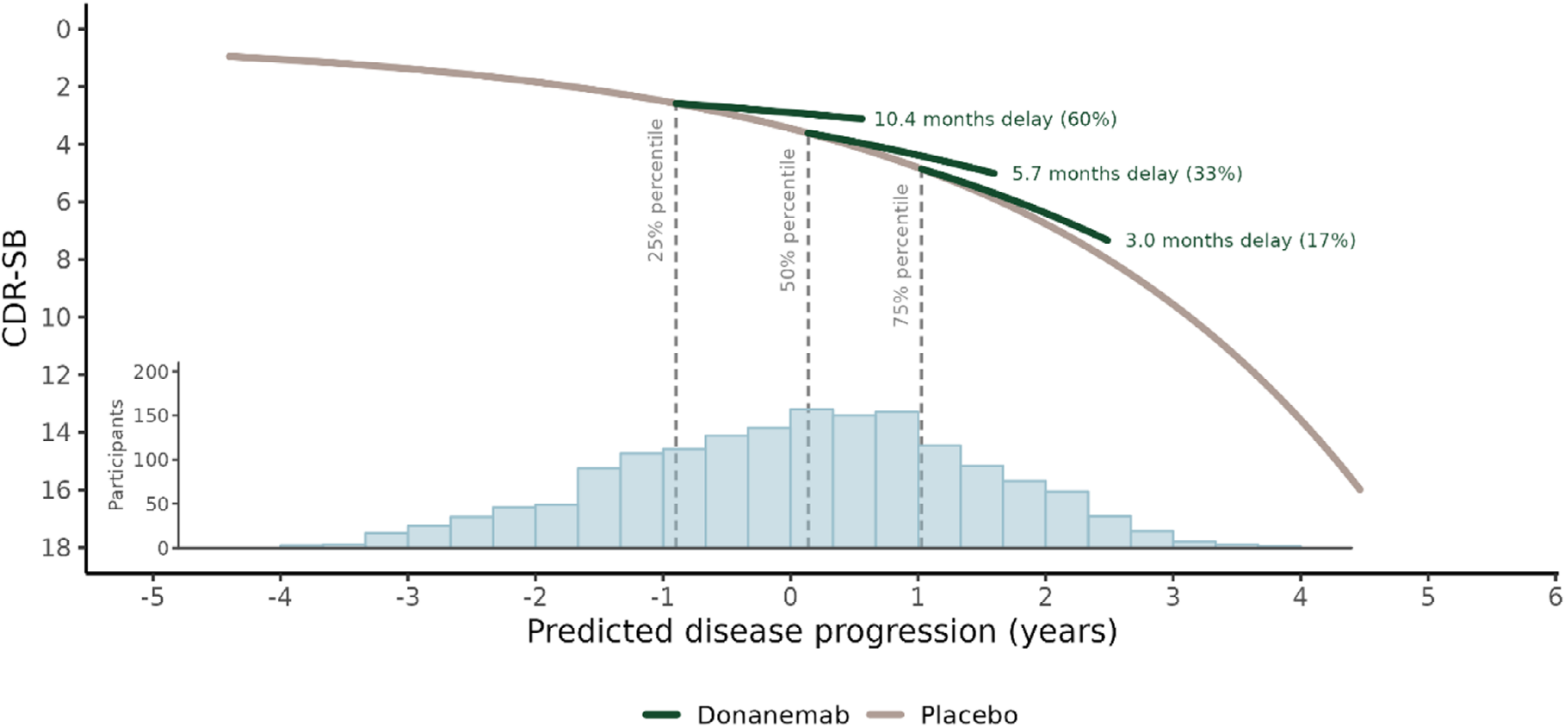
Estimated stage‐dependent treatment effects over 76‐week treatment period. A latent‐time mixed‐effects model was fitted on longitudinal trajectories of CDR‐SB scores, staging participants relative to each other on a predicted disease progression time scale. The treatment effect of donanemab was modeled as proportional time saving (green solid lines), depending on a participant's baseline predicted disease progression. This allows exploration of donanemab efficacy when initiating treatment at different times along the predicted disease progression timeline. The baseline distribution of predicted disease progression is shown in histogram (light blue), and the estimated effect of treatment initiation is illustrated at the 25^th^, 50^th^, and 75^th^ percentiles of the baseline predicted disease progression distribution. CDR‐SB, Clinical Dementia Rating Scale Sum of Boxes.

The latent‐time disease progression model found a significant relationship between baseline predicted disease progression and donanemab‐mediated time saving (*p* < 0.0001, Supplement 4) and suggests that initiating treatment at an earlier predicted disease progression was associated with greater time savings (Figure 2). Treatment at the 25^th^, 50^th^, or 75^th^ percentiles of the baseline predicted disease progression distribution was associated with a delay of disease progression by 60% (95% confidence interval [CI]: 50%, 70%), 33% (95% CI: 26%, 39%), and 17% (95% CI: 11%, 23%), respectively (Figure 2, Supplement 4, eTable 3, and eFigure 2). Notably, predicted disease progression demonstrated significant correlations with baseline tau PET (Spearman's *ρ* = 0.35, *p* < 0.0001) and baseline plasma P‐tau217 levels (Spearman's *ρ* = 0.27, *p* < 0.0001), but not amyloid PET (Spearman's *ρ* = ‐0.02, *p* = 0.36) as shown in eFigure 3 in Supplement 4.

**FIGURE 3 alz71577-fig-0003:**
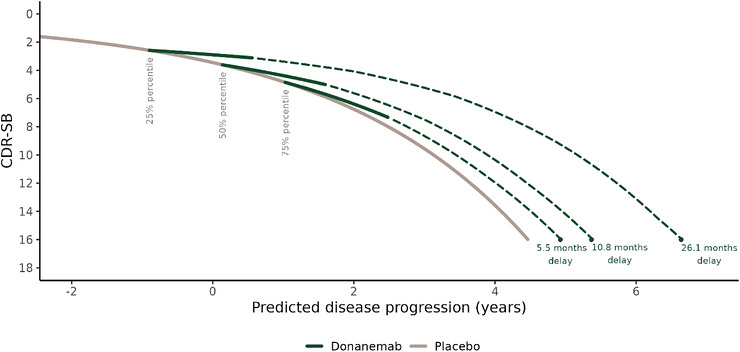
Modeling long‐term benefit of donanemab and delay to severe dementia. Long‐term trajectories of donanemab‐mediated delays (green dashed lines) of severe dementia (defined as CDR‐SB = 16) versus the placebo‐equivalent predicted disease progression (solid gray line) were extrapolated using a latent‐time disease progression model of CDR‐SB scores. The estimated effects of treatment initiation at the 25^th^, 50^th^, or 75^th^ percentiles of the baseline predicted disease progression distribution are illustrated. This extrapolation scenario corresponds to a fading‐slowing scenario where the time savings estimate is updated every 76 weeks since baseline, based on placebo‐equivalent predicted disease progression reached after every 76‐week period. CDR‐SB, Clinical Dementia Rating Scale Sum of Boxes.

Finally, Figure 3 illustrates long‐term extrapolation trajectories from the model, assuming diminishing accumulated time savings, where the proportional time saving estimate was updated every 76 weeks post‐baseline. According to this model estimate of the placebo‐equivalent predicted disease progression, the delays to severe dementia (defined as CDR‐SB = 16)^7^ were 26.1 months (25^th^ percentile), 10.8 months (50^th^ percentile), and 5.5 months (75^th^ percentile), corresponding to relative delays in time to severe dementia of 41%, 21%, and 13%. This extrapolation corresponds to a previously described stage‐dependent fading‐slowing scenario.^7^

## 4 DISCUSSION

The analyses presented here are complementary to the primary efficacy analyses of TRAILBLAZER‐ALZ 2 which used linear mixed models to estimate mean integrated Alzheimer's Disease Rating Scale (iADRS) and CDR‐SB change by treatment group over the 76‐week trial^2^. Those analyses remain the primary evidence for the average treatment effect in the recruited population. The post‐hoc tau‐stratified and latent‐time analyses presented here characterize how the magnitude of slowing varies with baseline biomarker levels and disease stage at treatment initiation.

These post‐hoc analyses found that donanemab treatment slowed clinical decline in participants with early symptomatic AD as defined by baseline tau PET, plasma P‐tau217 levels, or predicted disease progression. The greatest efficacy was consistently observed in those treated at an earlier disease stage. Subpopulation analyses found that donanemab treatment in participants with mild cognitive impairment and low‐to‐medium tau levels (i.e., those in the lowest plasma P‐tau217 tercile) led to a 46% reduction in decline compared to placebo^2^, as measured by CDR‐SB over 76 weeks, versus 29%^2^ in the overall population. These results indicate that donanemab treatment may effectively slow disease progression in people with a range of baseline clinical and pathological severities across the early symptomatic AD spectrum.

Relatively greater disease slowing in participants with a lower tau burden is biologically plausible, as advanced disease stages are associated with more amyloid‐independent processes contributing to decline.^8^, ^9^ Amyloid plaques accelerate the spread of tau; once tau levels are high, amyloid‐independent processes can drive neuronal damage, neuroinflammation, and further clinical decline.^10^

Recent studies have confirmed a sequential worsening of tau PET and plasma P‐tau217, followed by clinical impairment and dementia, along the AD continuum.^11^ Our study indicates that baseline tau levels impact clinical response to donanemab treatment, across both biomarkers associated with AD pathology. Although no guidelines definitively define disease stages using tau PET or plasma P‐tau217, our analyses demonstrate that patients with a quantitatively lower burden may benefit more from donanemab treatment. The association of P‐tau217 levels with treatment response was similar to that of tau PET, and tracked predicted disease progression almost as well, indicating the potential clinical utility of this more practical biomarker. Recent work has indicated that P‐tau217 is independently associated with both amyloid and tau burden, and may mediate the association of amyloid with tau, demonstrating a role in the underlying pathobiology of AD.^12^ In the TRAILBLAZER‐ALZ population, where the baseline amyloid burden was substantial^13^ and amyloid PET may have already reached a plateau in many participants,^8^ plasma P‐tau217 was more strongly correlated with tau PET than amyloid PET.^13^ Consistent with our findings, earlier initiation of lecanemab treatment has shown benefits in no‐tau/low‐tau and low‐amyloid patient populations at early stages of AD.^14^, [Fig alz71577-fig-0001], [Fig alz71577-fig-0002], [Fig alz71577-fig-0003]

Further studies are needed to expand on these findings and address the following limitations. First, the analyses were conducted post hoc, while the clinical implementation of blood‐based biomarkers for predicting the benefits of donanemab treatment requires prospective validation. Nevertheless, it is promising that plasma P‐tau217 levels may have suitable predictive capabilities, given the greater practicality of blood‐based biomarkers than neuroimaging. Second, our disease progression model requires constraints to be estimable; we used a parametric mean curve and assumed that the treatment effect manifests as proportional time saving, using a quadratic relation between the predicted disease stages and the degree of donanemab‐mediated slowing. Third, our disease progression model assumed a common mean trajectory of decline across participants, but the shape of the trajectory of decline may be affected by participant characteristics such as age.^15^ It is important to note that the model is designed to estimate population‐level dynamics rather than predict individual patient outcomes or inform patient‐specific treatment decisions; its primary objective is to identify those patients who are, on average, likely to derive the greatest benefit. Finally, the analysis of long‐term trajectories used population‐level results to extrapolate beyond the trial evidence of any single participant and should therefore be considered exploratory.

As with other non‐communicable diseases, addressing the key pathophysiological drivers of AD at an early stage of clinical disease may be most beneficial for slowing disease progression.

## CONFLICT OF INTEREST STATEMENT

Dr. Lars L. Raket; Dr. Ming Lu; Dr. Cynthia D. Evans; Dr. Jennifer A. Zimmer; Dr. JonDavid Sparks; Dr. Emily C. Collins; Dr. Sergey Shcherbinin; Dr. Hong Wang, Dr. Emel Serap Monkul Nery, Dr. Stephane Epelbaum, Dr. Grazia Dell'Agnello; Dr. Dawn A. Brooks; Dr. John R. Sims; and Dr. Mark A. Mintun reported being employees of and shareholders in Eli Lilly and Company. Author disclosures are available in the Supporting Information.

## CONSENT STATEMENT

All human subjects provided informed consent.

## Supporting information




Supporting Information

Supporting Information

## Data Availability

Lilly provides access to all individual participant data collected during the trial, after anonymization, with the exception of pharmacokinetic or genetic data. Data are available to request six months after the indication studied has been approved in the US and EU and after primary publication acceptance, whichever is later. No expiration date of data requests is currently set once data are made available. Access is provided after a proposal has been approved by an independent review committee identified for this purpose and after receipt of a signed data sharing agreement. Data and documents, including the study protocol, statistical analysis plan, clinical study report, blank or annotated case report forms, will be provided in a secure data sharing environment. For details on submitting a request, see the instructions provided at www.vivli.org.
