# Supplementary material for: Donanemab treatment effect by baseline tau burden and disease severity: Observations from the TRAILBLAZER‐ALZ 2 trial
Source: Alzheimers Dement. 2026 Jun 11;22(6):e71577. doi: 10.1002/alz.71577 (PMC13254682; doi:10.1002/alz.71577)
Supplement: Supplementary file 1 — Supporting Information [file ALZ-22-e71577-s001.docx]

Table of Contents

[Supplement 1: Latent-time Disease Progression Model (eMethods) 2](#_Toc228456236)

[Supplement 2: Patient Population 3](#_Toc228456237)

[eFigure 1. CONSORT Diagram 3](#_Toc228456238)

[Supplement 3: Baseline Demographics and Clinical Characteristics 4](#_Toc228456239)

[eTable 1. Baseline Demographics and Clinical Characteristics in the Low/Medium Tau Population 4](#_Toc228456240)

[eTable 2. Baseline Demographics and Clinical Characteristics in the High Tau Population 6](#_Toc228456241)

[Supplement 4: Latent-time Disease Progression Model Confidence Intervals and Test for Time-varying Treatment Effect 8](#_Toc228456242)

[eTable 3. Parameter estimates for latent-time disease progression model 8](#_Toc228456243)

[eFigure 2. Estimated Stage-dependent Treatment Effects Over 76-week Treatment Period Along with Marginal 95% Confidence Bands for Treatment Effects 9](#_Toc228456244)

[eFigure 3. Baseline Biomarkers Along Predicted Disease Progression 10](#_Toc228456245)

[Supplementary References 11](#_Toc228456246)

# Supplement 1: Latent-time Disease Progression Model (eMethods)

We used a latent-time disease progression model similar to previously published disease progression models.^1-3^ The core idea of this modeling framework is that disease progression on a disease-related measure such as a clinical scale follows a typical pattern across the population modeled by a continuous trajectory $\theta$ that describes how the clinical scale scores change with disease progression. The observed longitudinal data of the same clinical scale for a subject is then matched to the typical trajectory $\theta$ through a latent time variable that describes the patient’s disease stage, that is, for subject $i$, the latent disease stage $s_{i}$ corresponds to the time shift that makes the baseline clinical scale score corresponds to $\theta\left( s_{i} \right).$

We modeled CDR-SB scores using latent-time models, where the latent time shifts were modeled as random effects. We modeled donanemab treatment effects as a fixed effect describing the proportional time saving of disease progression that were allowed to depend on the baseline disease stage at treatment initiation. The model can be described as follows. Let $y_{\mathrm{ij}}$ denote subject *i*’s observed CDR-SB score at the *j*th visit happening $t_{\mathrm{ij}}$ years after baseline. We modeled the outcomes as

$$y_{\mathrm{ij}}=\theta\left( {\left( 1-\text{dona}_{i} \right)\cdot t}_{\mathrm{ij}}+s_{i} \right)+\varepsilon_{\mathrm{ij}}$$

where

- $\theta$ is a five-parameter logistic function

$$\theta\left( t \right)=A+\frac{K-A}{\left( 1+\exp\left( -B\cdot(t+s) \right) \right)^{v}}$$

- $\text{dona}_{i}$ is the treatment effect that models how donanemab treatment changes the rate of progression ($\text{dona}_{i}=0$ for subjects in the placebo arm), which was modelled as a quadratic function of latent disease time at initiation:

$$\text{dona}_{i}=\left\{ \begin{aligned} 0 \text{if treatment}\left( i \right)=\text{placebo, } \\ \beta_{0}+\beta_{1}s_{i}+\beta_{2}s_{i}^{2} \text{if treatment}\left( i \right)=\text{donanemab} \end{aligned} \right.$$

- $s_{i}$ is the latent disease stage of subject $i$ at baseline, $s_{i}\sim N\left( 0, \sigma_{s}^{2} \right),$
- $\varepsilon_{\mathrm{ij}}$ is the residual error, which is assumed to be independent $\varepsilon_{\mathrm{ij}}\sim N\left( 0,\sigma^{2} \right)$.

The model was implemented in R and C++ using the TMB package^3^ that utilizes automatic differentiation and Laplace approximations for computing the likelihood function. All fixed effects were estimated using maximum likelihood estimation.

# Supplement 2: Patient Population

## eFigure 1. CONSORT Diagram

**
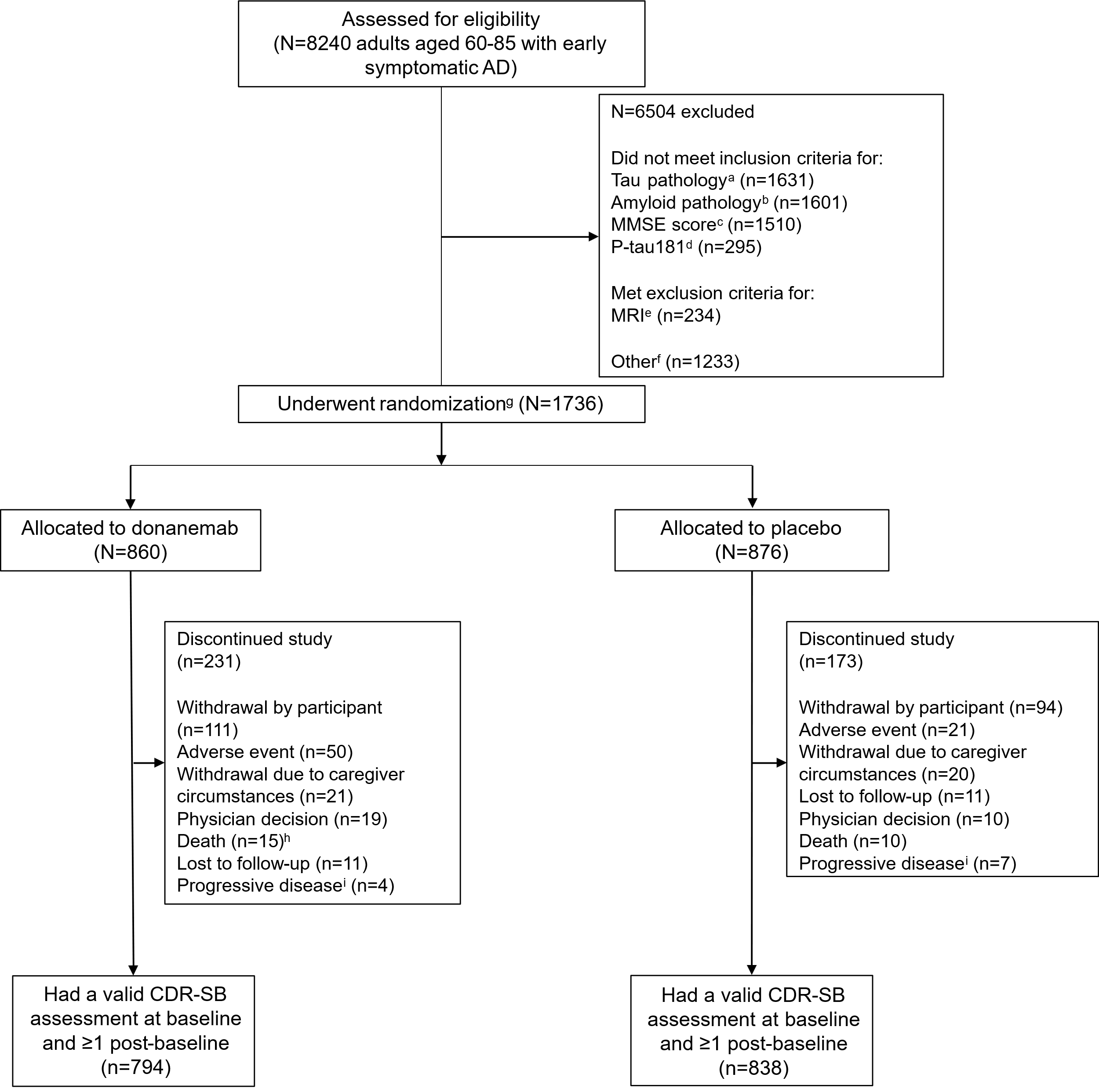
**

Abbreviations: AD, Alzheimer’s disease; CDR-SB, Clinical Dementia Rating Scale Sum of Boxes; MMSE, Mini-Mental State Exam; PET, positron emission tomography; p-tau181, phosphorylated tau 181

^a^Inclusion criteria for tau pathology: low/medium or high tau indicated by standardized uptake value ratio >1.10 or positive visual read assessed by ^18^F-flortaucipir PET imaging. ^b^Inclusion criteria for amyloid pathology (≥37 Centiloids) assessed with ^18^F-florbetapir or ^18^F-florbetaben PET. ^c^Inclusion criteria for MMSE: score of 20 to 28. ^d^P-tau181 screening criterion was not implemented for the entire trial duration (eMethods in Supplement 3 of ^4^). ^e^Exclusion criteria for MRI include presence of amyloid-related imaging abnormalities of edema/effusion, >4 cerebral microhemorrhages, >1 area of superficial siderosis, and any intracerebral hemorrhage >1 cm or severe white matter disease. ^f^Summary of other screen failure can be found in eTable 3 in Supplement 3 of ^4^ (lists reason if ≥20 participants). ^g^Stratified by baseline tau categorization and enrolling sites. ^h^One additional death occurred after treatment completion and in the follow-up period. ^i^Alzheimer disease progression to a degree prompting study discontinuation, per investigator judgment.

# Supplement 3: Baseline Demographics and Clinical Characteristics

## eTable 1. Baseline Demographics and Clinical Characteristics in the Low/Medium Tau Population

|  | **Placebo**  **(N=594)** | **Donanemab**  **(N=588)** | **Total**  **(N=1182)** |
| --- | --- | --- | --- |
| **Age, mean [SD], y** | 74.26 [5.78] | 74.31 [5.71] | 74.28 [5.74] |
| **Age category,  No. (%)** |  |  |  |
| <65 | 34 (5.7) | 31 (5.3) | 65 (5.5) |
| 65-74 | 256 (43.1) | 265 (45.1) | 521 (44.1) |
| ≥75 | 304 (51.2) | 292 (49.7) | 596 (50.4) |
| **Sex, No. (%)** |  |  |  |
| Female | 321 (54.0) | 325 (55.3) | 646 (54.7) |
| Male | 273 (46.0) | 263 (44.7) | 536 (45.3) |
| **Race, No. (%)** |  |  |  |
| American Indian or Alaska Native | 0 | 1 (0.2) | 1 (0.1) |
| Asian | 38 (6.4) | 48 (8.2) | 86 (7.3) |
| Black or African American | 17 (2.9) | 17 (2.9) | 34 (2.9) |
| White | 539 (90.7) | 522 (88.8) | 1061 (89.8) |
| **Ethnicity, No. (%)^a^** |  |  |  |
| Hispanic or Latino | 26 (6.3) | 24 (5.8) | 50 (6.0) |
| Not Hispanic or Latino | 390 (93.8) | 390 (94.2) | 780 (94.0) |
| Missing | 1 | 1 | 2 |
| **Time since symptom onset, mean [SD], y** | 3.79 [2.42] | 4.00 [2.63] | 3.89 [2.53] |
| Missing | 1 | 0 | 1 |
| **Time since AD diagnosis, mean [SD], y** | 1.35 [1.75] | 1.49 [1.83] | 1.42 [1.79] |
| Missing | 5 | 7 | 12 |
| ***APOE* ε4 status, No. (%)** |  |  |  |
| Carrier | 427 (72.3) | 421 (71.7) | 848 (72.0) |
| Non-carrier | 164 (27.7) | 166 (28.3) | 330 (28.0) |
| Missing | 3 | 1 | 4 |
| **Baseline CDR-SB score, mean [SD]** | 3.69 [2.04] | 3.74 [2.10] | 3.71 [2.07] |
| Missing | 3 | 8 | 11 |
| **Baseline CDR global score, No. (%)** |  |  |  |
| 0 | 3 (0.5) | 2 (0.3) | 5 (0.4) |
| 0.5 | 387 (65.5) | 382 (65.9) | 769 (65.7) |
| 1 | 185 (31.3) | 177 (30.5) | 362 (30.9) |
| 2 | 16 (2.7) | 19 (3.3) | 35 (3.0) |
| Missing | 3 | 8 | 11 |
| **Baseline MMSE total score^b^, mean [SD]** | 22.81 [3.77] | 23.05 [3.64] | 22.93 [3.71] |
| Missing | 0 | 5 | 5 |
| **Screening tau MUBADA SUVr, mean [SD]** | 1.21 [0.13] | 1.21 [0.12] | 1.21 [0.12] |
| **Screening amyloid centiloid, mean [SD]** | 100.92 [35.13] | 102.36 [34.74] | 101.64 [34.93] |

Abbreviations: AD, Alzheimer’s disease; *APOE*, apolipoprotein E; CDR, Clinical Dementia Rating Scale; MMSE, Mini-Mental State Exam; MUBADA multi-block barycentric discriminant analysis; N, number of randomized subjects; SB, Sum of Boxes; SUVr, standardized uptake value ratio.

^a^Responses only from US/Puerto Rico sites. n=416 placebo, n=414 donanemab, n=830 total

^b^Last non-missing MMSE prior to or on start of study treatment.

In variables with missing data, number of subjects with non-missing data was used as denominator.

## eTable 2. Baseline Demographics and Clinical Characteristics in the High Tau Population

|  | **Placebo**  **(N=281)** | **Donanemab**  **(N=271)** | **Total**  **(N=552)** |
| --- | --- | --- | --- |
| **Age, mean [SD], y** | 70.47 [6.31] | 70.10 [6.15] | 70.29 [6.22] |
| **Age category, No. (%)** |  |  |  |
| <65 | 54 (19.2) | 57 (21.0) | 111 (20.1) |
| 65-74 | 145 (51.6) | 148 (54.6) | 293 (53.1) |
| ≥75 | 82 (29.2) | 66 (24.4) | 148 (26.8) |
| **Sex, No. (%)** |  |  |  |
| Female | 181 (64.4) | 167 (61.6) | 348 (63.0) |
| Male | 100 (35.6) | 104 (38.4) | 204 (37.0) |
| **Race, No. (%)** |  |  |  |
| American Indian or Alaska Native | 0 | 1 (0.4) | 1 (0.2) |
| Asian | 9 (3.2) | 9 (3.3) | 18 (3.3) |
| Black or African American | 4 (1.4) | 2 (0.7) | 6 (1.1) |
| White | 267 (95.0) | 258 (95.6) | 525 (95.3) |
| Multiple | 1 (0.4) | 0 | 1 (0.2) |
| Missing | 0 | 1 | 1 |
| **Ethnicity, No. (%)^a^** |  |  |  |
| Hispanic or Latino | 10 (4.7) | 11 (5.4) | 21 (5.0) |
| Not Hispanic or Latino | 203 (95.3) | 192 (94.6) | 395 (95.0) |
| Missing | 1 | 1 | 2 |
| **Time since symptom onset, mean [SD], y** | 4.05 [2.24] | 3.82 [2.32] | 3.94 [2.28] |
| **Time since AD diagnosis, mean [SD], y** | 1.55 [1.80] | 1.50 [1.67] | 1.53 [1.74] |
| Missing | 2 | 1 | 3 |
| ***APOE* ε4 status, No. (%)** |  |  |  |
| Carrier | 193 (68.9) | 176 (65.4) | 369 (67.2) |
| Non-carrier | 87 (31.1) | 93 (34.6) | 180 (32.8) |
| Missing | 1 | 2 | 3 |
| **Baseline CDR-SB score, mean [SD]** | 4.42 [2.03] | 4.43 [1.96] | 4.42 [1.99] |
| Missing | 4 | 7 | 11 |
| **Baseline CDR global score, mean [SD]** |  |  |  |
| 0 | 1 (0.4) | 0 | 1 (0.2) |
| 0.5 | 144 (52.0) | 132 (50.0) | 276 (51.0) |
| 1 | 123 (44.4) | 126 (47.7) | 249 (46.0) |
| 2 | 9 (3.2) | 6 (2.3) | 15 (2.8) |
| Missing | 4 | 7 | 11 |
| **Baseline MMSE total score^b^, mean [SD]** | 20.76 [3.85] | 21.08 [3.94] | 20.91 [3.90] |
| Missing | 5 | 5 | 10 |
| **Screening tau MUBADA SUVr, mean [SD]** | 1.70 [0.20] | 1.68 [0.17] | 1.69 [0.18] |
| Missing | 57 | 43 | 100 |
| **Screening amyloid centiloid, mean [SD]** | 103.11 [33.14] | 106.01 [33.83] | 104.53 [33.48] |

Abbreviations: AD, Alzheimer’s disease; *APOE*, apolipoprotein E; CDR, Clinical Dementia Rating Scale; MMSE, Mini-Mental State Exam; MUBADA, multi-block barycentric discriminant analysis; N, number of randomized subjects; SB, Sum of Boxes; SUVr, standardized uptake value ratio.

^a^Responses only from US/Puerto Rico sites. n=213 placebo, n=203 donanemab, n=416 total

^b^Last non-missing MMSE prior to or on start of study treatment.

In variables with missing data, number of subjects with non-missing data was used as denominator.

# Supplement 4: Latent-time Disease Progression Model Confidence Intervals and Test for Time-varying Treatment Effect

**Test of Time-varying Treatment Effect**

Null hypothesis ${H_{0}:\beta}_{1}=\beta_{2}=0$ tested with likelihood ratio test. $\text{LR}=87.81, \text{df}=2, p=8.5\cdot{10}^{-20}$

Cross-validated Prediction Interval Coverage

To assess calibration of the latent-time model, we performed 10-fold cross-validation stratified by treatment arm. For each fold, the model was refit on 90% of subjects. Each held-out subject's disease stage was estimated from their baseline CDR-SB alone via empirical Bayes, and the full follow-up trajectory was predicted. A 95% prediction interval was constructed at each visit, accounting for residual variance and disease stage estimation uncertainty propagated through the nonlinear progression function via the delta method. Of 8,456 follow-up observations, 94.0% fell within the 95% prediction intervals (95.3% for donanemab, 92.8% for placebo), confirming well-calibrated uncertainty quantification.

Confidence Interval Computation

Confidence intervals for variance components (latent shift and residual standard deviations) and treatment slowing estimates at the 25%, 50%, and 75% percentiles of predicted disease stage were obtained by profile likelihood applied to the Laplace-approximated marginal log-likelihood.

For the slowing estimates, which are linear combinations of the polynomial slowing coefficients evaluated at specific shift values, profile likelihood was computed over the corresponding linear combination of parameters.

## eTable 3. Parameter estimates for latent-time disease progression model

| Parameter | Estimate | 95% Confidence Interval^a^ |
| --- | --- | --- |
| Latent time shift standard deviation $\boldsymbol{\sigma}_{\boldsymbol{s}}$ | 1.47 | (1.39, 1.55) |
| Residual noise standard deviation $\boldsymbol{\sigma}$ | 1.08 | (1.07, 1.10) |
| Delay in disease progression |  |  |
| At 25^th^ percentile of baseline | 60% | (50%, 70%) |
| At 50^th^ percentile of baseline | 33% | (26%, 39%) |
| At 75^th^ percentile of baseline | 17% | (11%, 23%) |

Abbreviation: PDP, predicted disease progression

^a^Confidence intervals obtained by profiling the likelihood function.

## eFigure 2. Estimated Stage-dependent Treatment Effects Over 76-week Treatment Period Along with Marginal 95% Confidence Bands^a^ for Treatment Effects


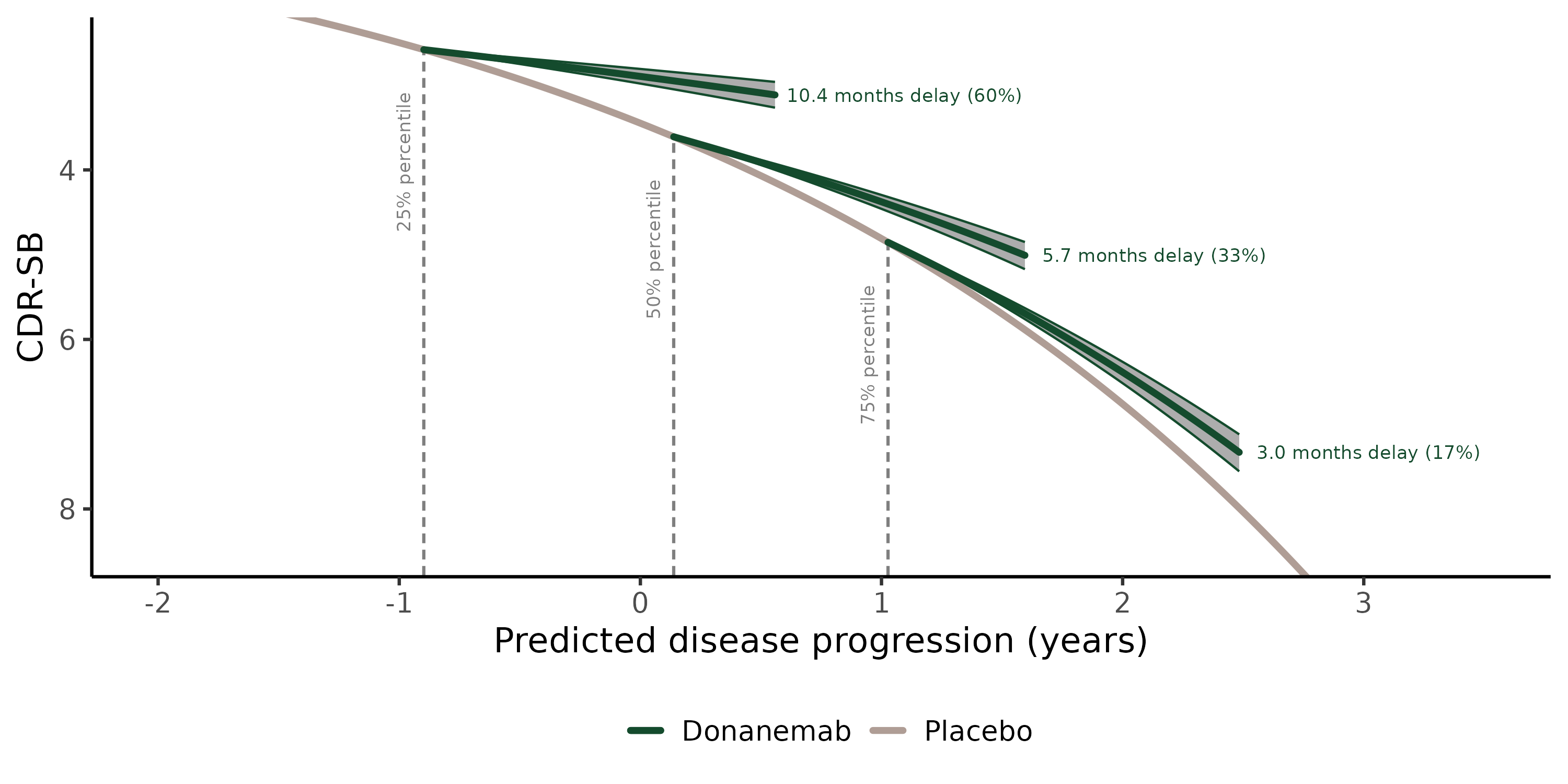


Abbreviation: CDR-SB, Clinical Dementia Rating Scale Sum of Boxes ^a^ The 95% shaded band represents a marginal confidence band for the donanemab-vs.-placebo treatment-effect contrast at each predicted disease progression percentile^.^

## eFigure 3. Baseline Biomarkers Along Predicted Disease Progression


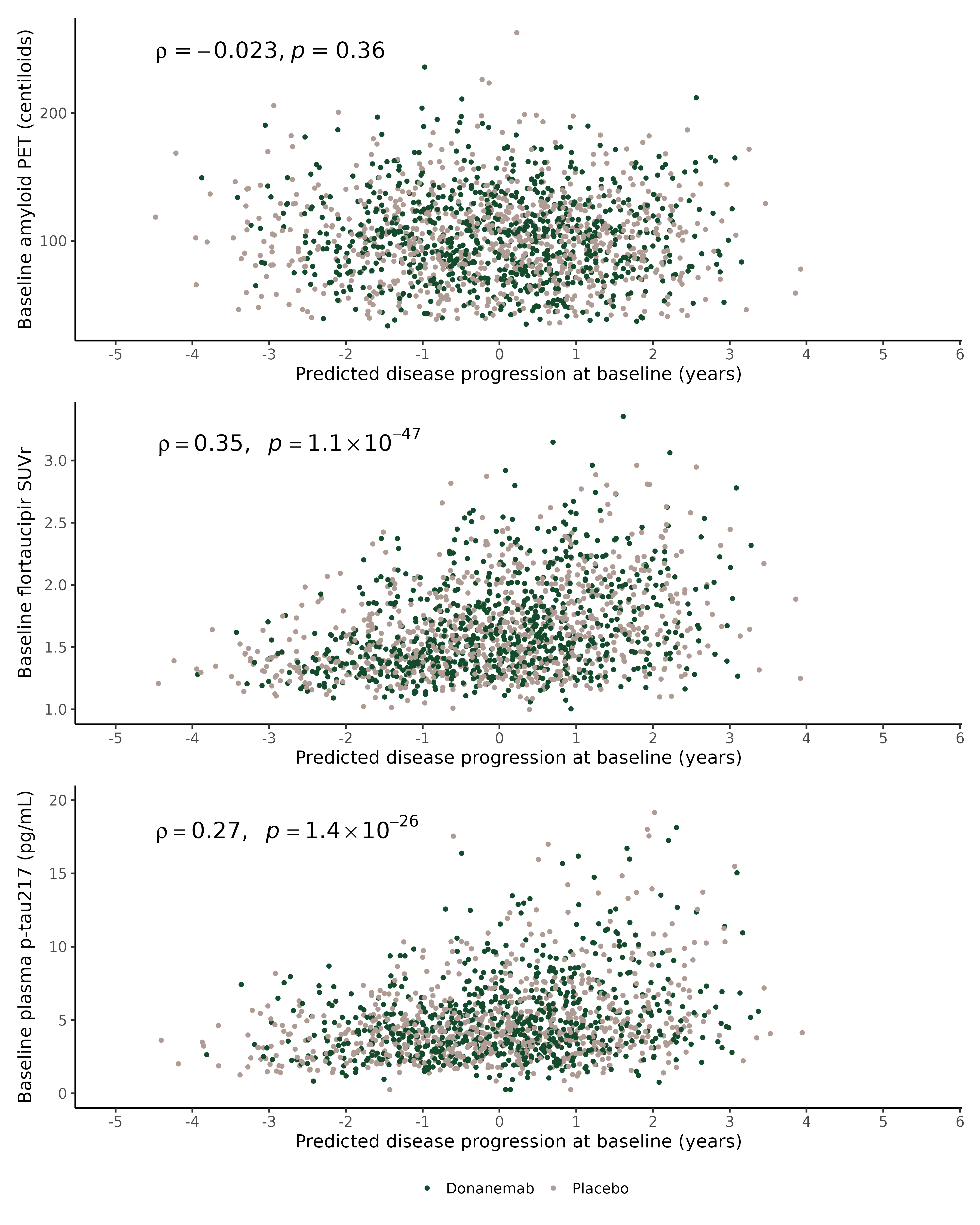


Abbreviations: PET, positron emission tomography; SUVr, standardized uptake value ratio. ρ denotes Spearman correlations.

Note: 41 plasma p-tau217 observations with values above 20 pg/mL are not shown in the figure.

# Supplementary References

1. Kühnel L, Berger AK, Markussen B, Raket LL. Simultaneous modeling of Alzheimer's Disease progression via multiple cognitive scales. *Stat Med*. 2021;40(14):3251-3266. doi:10.1002/sim.8932

2. Raket LL. Statistical disease progression modeling in Alzheimer Disease. *Front Big Data*. 2020;3:24. doi:10.3389/fdata.2020.00024

3. Kristensen K, Nielsen A, Berg CW, Skaug H, Bell BM. TMB: Automatic differentiation and laplace approximation. *J Stat Softw*. 2016;70(5):1 - 21. doi:10.18637/jss.v070.i05
4. Sims JR, Zimmer JA, Evans CD, *et al*. Donanemab in early symtomatic Alzheimer disease: The TRAILBLAZER-ALZ2 randomized clinical trial. *JAMA*. 2023;330(6):512-27. doi:10.1001/jama.2023.13239.
